# Standing up for Subjectivity in the Assessment of Competencies

**DOI:** 10.3205/zma001175

**Published:** 2018-08-15

**Authors:** Thomas Rotthoff

**Affiliations:** 1 Heinrich-Heine-University Düsseldorf, Medical Faculty, Office of the dean of studies, Düsseldorf, Germany

## Competence-orientation for Boosting Employability

Over the past decade, “competency-based” and “outcome-based” education have almost become a paradigm with the status of a “god term” in medical education, training and further education [1]. Formerly education, knowledge and qualification were the goals of higher education, today the development of competencies and key competencies is generally given priority [2]. The development of Competency-Based Education (CBE) is essentially based on political motives rather than scientific evidence [3], [4], [5], [6]. The political motives are closely linked to the debate on “employability” [2]. Development of competence is thus geared to the operational exigencies of social practice [7]. Today, the quality of education is determined more strongly by whether knowledge is not only appropriated and reproduced, but that knowledge can also be competently applied in solving new problems [7]. These motives appear to be particularly congenial to medical studies with their defined professional goal, since the Licensure Act requires the training of doctors capable of independent practice and responsibility. However, critics see CBE as poorly suited to highly skilled professions in particular such as doctors because they require highly complex skills such as analysis, judgment and reflection, professionalism and empathy. These – according to the critics – cannot be adequately achieved with the predominant methods of didactic learning objectives prevalent so far with CBE [3], [8], [9]. 

## From Qualification to Competence

Therefore for a purposeful and critical discussion, it is worthwhile first of all to hone the notions of competence, key competence, qualification, knowledge and skills used in the education sciences beyond medicine. According to Arnold (1997), the term “qualification” is fact-centered and limited to immediate activity-related knowledge and skills, whereas competence is also value- or attitude-oriented, extends to the whole person and focuses on the developmental possibilities and ability to act of the individual [7]. As so-called key competencies, the dimensions of “subject competence”, “methodological competence”, “social competence” as well as “personal” or “self-competence” have become widespread and established nationally and internationally despite the lack of theoretical justification [2]. Various conceptualizations of the concept of competence have come closer in recent times and have, inter alia, the similarities that competence and key competencies relate to the ability to perform tasks of high complexity and that acquired competencies are not directly, i.e. one to one without relearning and adaptation processes, applicable to in new situations. The complexity of key competencies suggests that they can only be deduced from activity i.e. performance [2], [7]. The English-language definition of professional (medical) competence listed in the NKLM also clearly illustrates the involvement of the entire person and goes beyond qualification as an acquisition of knowledge and skills: “Professional competence is the habitual and judicious use of communication, knowledge, technical skills, clinical reasoning, emotions, values and reflections in daily practice for the benefit of the individual and community being served” [10]. 

Medical work is concerned with the ability to integrate different competencies for optimal patient care according to the situation [11]. It is precisely this content and context specificity of clinical action that is cited as contradicting isolated training in different competencies, as students can perform well in one case and poorly in another with a different context [12], i.e. they cannot directly apply acquired competencies to new situations without using adaptation processes [2].

The central approach of competence-oriented education in medicine has so far been based on the assumption that professional roles can be broken down and operationalized into individual elements of defined knowledge or skills, which – acquired separately – then lead to comprehensive competence [3]. This procedure has not yet been adequately supported by empirical evidence and indeed is critically discussed in the current literature because the entire competence is more than the sum of individually successfully completed tasks in terms of knowledge or skills [3], [9], [13], [14], [15]. Competency and key competencies differ from knowledge or simple skills in fulfilling tasks of high complexity [2]. 

## Competencies reveal themselves through performance in the medical work place

Although isolated examinations of knowledge, skills or attitudes measure important prerequisites for competence, they do not constitute tests of competence in the true sense! Despite many years of development and implementation of competence-based curricula, in the estimation of Hodges & Lingard sophisticated test concepts for the measurement of competencies still only scratch the surface [1]. Competence-oriented training models are even attested to a lack of reliable examination formats and higher-level examination strategies [6]. If so, what has led to CBE becoming today’s paradigm in medical education? Are we likely to be subject to a spectator effect in which the opinion of an individual is all the more influenced the more distinctive the opinion (including by prominent voices) is expressed on a topic [16]? We should not look at the matter quite so pessimistically, and focus more on the definition of competence described above when assessing competencies. Harris and Keller already pointed out in 1976 that *“the major development effort in competency-based education should not lie in the design of instructional materials but in the design of appropriate performance assessments. Furthermore, institutions should not commit themselves to competency-based curricula unless they possess means to directly assess students’ performance.“* [17]. Thus, if competencies and key competencies can only be revealed and therefore tested by performance in action [2], CBE should be geared specifically to the action requirements of medical practice, which integrate different competencies and roles with variable importance. For medical students, this means creating more situations in the curriculum, in which medical practice (performance) is trained and observed in varying degrees of complexity in real and realistic situations. Undoubtedly, this presents a challenge with large numbers of students. The development of “Entrustable Professional Activities” (EPA) [18] shows a possible way forward. Such EPAs can be held at different granularities. In earlier stages of study, simple diagnostic and therapeutic activities can already be taught under supervision and tested in conjunction with other competencies. In the Practical Year the activities can become more extensive and include for example, dealing with the care of a patient with a chronic illness or the complete discharge management of a patient. Performance is thus trainable in the course of study with different levels of complexity. But how can performance be tested? 

## Testing performance to capture competencies

The current discussion about the testing of competencies is strongly influenced by the aspiration to capture competencies as objectively, reliably and validly as possible in order to best meet test quality criteria. In the past, attempts to capture more complex competencies with objective tools often fell short [9], [19]. Are test quality criteria for assessing competencies even crucial? The answer depends on the goal we want to connect with CBE. In the current literature, the primary goal defined is a learning control for competence development, from which result first of all formative assessment of performance in the sense of an “assessment for learning” [20]. CBE should record the students’ development, provide feedback on the level of competence achieved (e.g. through feedback) and accompany the students in their learning process [21]. Only secondarily should CBE examinations aim to make summative decisions on competence or incompetence in the sense of an “assessment of learning” [20]. Let’s look first at the requirements for formative testing of performance. 

## Formative measurement of performance

The assessment of performance is seen as a decision-making process that is influenced by the interactions between people and the context in which testing takes place [22]. The changing contexts always require new relearning and adaptation processes [2], [7]. In the field of performance, competencies have to be examined that can not be captured by quantitative measurement methods for competence assessment [23]. Quantitative measurement methods reduce the concept of competence to qualification with knowledge and skills, which is why they are also considered unsuitable for the design of competence-oriented development processes in practice [23]. Today there is even an increasing agreement that for the assessment of development processes and competencies the personal assessment of an examiner plays an especially important role, although human assessment as the only measure of validity, of course, has its limitations [24]. Just as a diagnosis or treatment cannot be made on the basis of laboratory values alone, but also includes one’s own judgment with subjective considerations, intuition and ethical considerations [25], so the testing of competencies should include the judgment of the examiners and not exclusively be subject-related [26]. The act of entrusting at EPA is also largely based on subjective considerations, which are mainly judged on the basis of supervision. The act of entrusting is always accompanied by a degree of uncertainty.

Formative performance measurement requires less objectivity and reliability though a high validity for credible feedback. On the basis of Kane’s theoretical framework model for validity, apart from a large number of observations and a high variance of the observation conditions, the clear definition of the construct to be measured is also important [27]. The validation is then an assessment of the relationship between the interpretation of the test result and the plausibility of the conclusions derived from it [27]. Therefore for examiners to reach a valid judgment at least familiarity with the defined constructs (roles, competencies) is necessary, better still a reflected discussion, since competencies are also value- or attitude-oriented. 

Even though checklists or global rating scales attempt to standardize formative assessments, the assessment remains subjective and requires understanding and familiarity with the examination situation and the dimensions to be measured. How else can observations of examiners be validly transformed into scores? If the users are uncertain, the quality of the feedback on the competence level of the students and the validity of such performance measurements is very limited even with many observations and a wide variety of observation conditions. This is often reflected in the tendency for above-average ratings and a lack of use of point score assessment tools in the workplace, regardless of the type of scaling [28]. 

Validity is therefore the decisive factor for the assessment of performance in the sense of a credible feedback on the attained competence level and support of the learning process. However, if examinations are to be summative and have consequences for the continuation of studies, they must be fair. That requires objectivity and reliability. So let’s look at how these two dimensions can be applied to performance testing.

## Summative Measurement of Performance

Although focusing on the objectivity and standardization of an exam increases its reliability, it is particularly in the medical working environment that there is a risk of moving away from reality and an authentic examination scenario with changing contexts [22]. In the real work environment, it is therefore more difficult to achieve an objective and reliable performance test [29]. The more complex the examination situation and the more competencies that are to be integrated, the more difficult it becomes to chart an objective and reliable competence-specific examination [19]. From a test theory perspective a more complex integration of competencies in an examination also comes at the expense of the substantive validity of the individual competence [30]. Sometimes an objective competence or performance measurement is even considered impossible; because what is today regarded as the attribute of a good doctor was not 50 years ago [24]. The idea of what constitutes competence or competent medical action changes over time and is not a stable construct [31]. At best, there is current agreement on what a good doctor should be and based on this, we decide what is to be integrated into a curriculum and how it should be taught [25]. In doing so, we are shaped by an environment in which ideas come and go, influenced by political, social and economic ideas as well as the conditions of the time [31]. Competencies and roles are social and not objectively stable constructs, and decisions about competence or incompetence are ultimately based on expert opinions [19]. This seems to affirm the critics regarding the lack of suitable exam concepts for CBE. 

## Programmatic Assessment as a Solution?

The use of test methods combines the claim to obtain psychometrically substantiated statements about latent abilities and interesting characteristics of individuals, which are based on assumptions about relationships between the characteristic to be measured and the observed test behavior [32]. Due to the above-mentioned difficulties in measuring performance objectively, validly and reliably, the characteristic features and relationships of interest could be captured using a programmatic approach in the sense of an examination portfolio. Depending on the importance of the test, a different number of tests or observations could be combined to form an overall assessment [33]. For standardized tests on knowledge and skills this is easy to implement. For the use of non-standardized performance tests in the medicalworking environment, the meaningfulness of such a procedure depends crucially on the validity of these examinations and thus also on the examiners. Overall, a promising approach, which initially requires a certain change of perspective on competence-oriented testing.

### Ability evolves from learning 

Today when measuring performance we are very much focused on the observable **ability**, which should correspond to previously defined benchmarks such as outcomes, milestones and learning goals. From the observed behavior of the person conclusions are drawn on non-observable dispositions in the assumption that the person could only show this ability because they have acquired the corresponding dispositions (= competence) and not only shown the behavior once (= output), but can always generate it (= outcome) [34]. On the basis of the preceding explanations the correctness of this assumption regarding performance may be doubted, since different conceptualizations of the concept of competence come to the common realization that competence and key competencies describe the ability to perform tasks of high complexity and that acquired competencies cannot be applied directly, i.e. one to one without relearning and adaptation processes, in new situations [2]. This brings us to a previously unresolved problem of how performance should be measured summatively. We run the risk of investing our resources in the best possible standardization of exams or perfecting checklists and scales in order to find a solution to this problem and lose sight of the more crucial **learning process** for CBE. The effects of an outcome-oriented CBE on later performance on the job have not been sufficiently proven. Alternatively, it can also be hypothesized that a stronger focus, support, examination, reflection and exploration of the **learning processes** can ultimately lead to more “ability” in the sense of performance. During the learning process, competence dimensions such as emotions and values [10] can be integrated more strongly in the interaction between teachers and students. Although these dimensions are required in the outcomes, they are only rarely developed jointly with the students. Up to now the way to reach a goal or outcome is more or less left to the students themselves. 

## Competence orientation requires new approaches in faculty development

Greater consideration of the learning processes also requires a further development of the expectations and attitudes of the people in a faculty over competence-oriented education; especially if the individual examiner becomes more important. Certainly we all feel that this is easier said than done and that traditional patterns of medical culture can be an obstacle. We need new approaches to faculty development and new didactic formats for CBE, which will allow more for a serious exchange in the discussion of competencies. Therefore, faculty training should not be limited to individual assessment tool workshops, such as how to complete a checklist for a Mini-Clinical Examination [24]. The examiners should exchange and discuss their assessments and exam situations [24], [35]. Ultimately, the available testing tools are only as good as the people who use them. Medical action is always characterized by the risk of the actions and insecurities of the agents themselves [25]. We should also accept these uncertainties for competence-oriented assessment, and have more courage to accept subjectivity in measuring performance. The most important prerequisite for this is the high validity and credibility of these assessments in order to actually support the students in their learning process and the acquisition of competencies. An extension of the focus of attention on the process of actual learning, which has hitherto been very focused on outcomes, requires the teachers to intensify employment and reflection with the dimensions of competence and a mutual exchange of experiences within the faculties. So far, there are no established structural or didactic concepts for this. Only then does consideration of non-standardized performance tests in a programmatic audit approach really make sense. 

## Competing interests

The author declares that he has no competing interests.
